# Recognition of G-quadruplex RNA by a crucial RNA methyltransferase component, METTL14

**DOI:** 10.1093/nar/gkab1211

**Published:** 2021-12-15

**Authors:** Atsuhiro Yoshida, Takanori Oyoshi, Akiyo Suda, Shiroh Futaki, Miki Imanishi

**Affiliations:** Institute for Chemical Research, Kyoto University, Uji, Kyoto 611-0011, Japan; Graduate School of Integrated Science and Technology, Shizuoka University, 836 Ohya, Suruga-ku, Shizuoka 422-8529, Japan; Institute for Chemical Research, Kyoto University, Uji, Kyoto 611-0011, Japan; Institute for Chemical Research, Kyoto University, Uji, Kyoto 611-0011, Japan; Institute for Chemical Research, Kyoto University, Uji, Kyoto 611-0011, Japan

## Abstract

*N*6-methyladenosine (m6A) is an important epitranscriptomic chemical modification that is mainly catalyzed by the METTL3/METTL14 RNA methyltransferase heterodimer. Although m6A is found at the consensus sequence of 5′-DRACH-3′ in various transcripts, the mechanism by which METTL3/METTL14 determines its target is unclear. This study aimed to clarify the RNA binding property of METTL3/METTL14. We found that the methyltransferase heterodimer itself has a binding preference for RNA G-quadruplex (rG4) structures, which are non-canonical four-stranded structures formed by G-rich sequences, via the METTL14 RGG repeats. Additionally, the methyltransferase heterodimer selectively methylated adenosines close to the rG4 sequences. These results suggest a possible process for direct recruitment of METTL3/METTL14 to specific methylation sites, especially near the G4-forming regions. This study is the first to report the RNA binding preference of the m6A writer complex for the rG4 structure and provides insights into the role of rG4 in epitranscriptomic regulation.

## Graphical Abstract

**Graphical Abstract:**
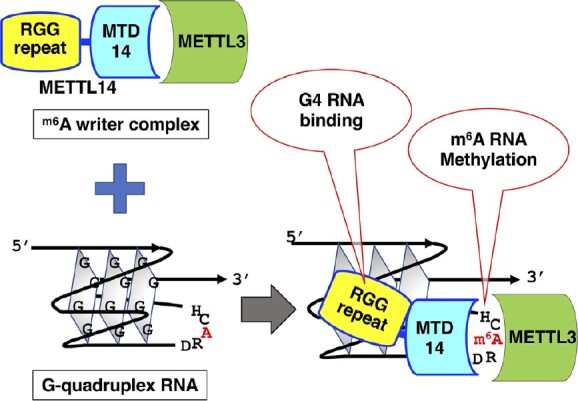
The RNA methyltransferase METTL3/METTL14 heterodimer was shown to preferentially bind to RNA G-quadruplex (rG4) structures and methylate adenosines close to rG4, providing insights into the role of rG4 in epitranscriptomic regulation.

## INTRODUCTION

*N*6-methyladenosine (m6A) modification is the most abundant mRNA modification and is also found in microRNAs, long non-coding RNAs, and viral RNAs. Because of the crucial roles of m6A modification in cell differentiation (1), carcinogenesis (2), circadian clock (3) and viral life cycles (4), it has been extensively studied in the fields of biology and chemical biology. The m6A modification is mainly catalyzed by the m6A writer complex composed of a methyltransferase-like (METTL)3/METTL14 heterodimer and additional adaptor proteins. METTL3 is the catalytic core. Although the methyltransferase domain (MTD) of METTL14 has no catalytic activity, METTL14 is essential for the methylation activity of METTL3, and it facilitates complex integrity and RNA binding (5–7). The m6A writer complex installs m6A at the consensus sequence 5′-DRACH-3′ (D = A, G, or U; R = A or G; H = A, C, or U) (8,9) but only a small fraction of the consensus sites are methylated. Elucidating the mechanism of target specificity of the m6A writer complex is necessary for understanding the biological roles of m6A and for designing targeted methyltransferases for site-specific manipulation of RNA methylation.

To date, both extrinsic and intrinsic factors have been proposed as the determinants of methylation of specific RNA regions by METTL3/METTL14 (10). Extrinsic factors include mediators such as RNA binding proteins, RNA polymerase II and H3K36me3 modification that interact with METTL3/METTL14 and recruit the m6A writer complex to specific RNA regions of nascent RNAs (11–16). Although these factors play a part in the target selectivity of the m6A writer complex, these interactions are not involved in all m6A modifications. In addition, the reported interactions were limited to cotranscriptional m6A modification in the nucleus, although METTL3/METTL14 also methylates the genomic RNA of positive-sense single-stranded RNA viruses whose replication occurs only in the cytoplasm (17,18). Thus, other factors must also be contributing to determine methylation target specificity. The intrinsic factors include local sequence features surrounding m6A, as suggested by sequence analysis in *Saccharomyces cerevisiae* (19). Recent bioinformatics analyses suggested co-localization of m6A and potential G-quadruplex (G4)-forming sequences in viral RNA (Zika virus (ZIKV) and human immunodeficiency virus (HIV)) and at human pre-mRNA intron splice sites (20,21). However, the intrinsic sequence preference of METTL3/METTL14 has not been experimentally verified.

G4s are non-canonical four-stranded structures comprising G-rich sequences in DNA and RNA, in which several guanine (G)-quartets formed by Hoogsteen hydrogen bonding are hydrophobically stacked. Potassium ions are known to stabilize G4 structures by coordinating between two G-quartets planes, whereas lithium ions do not show such stabilizing effects because of the small ion radius (22,23) (Supplementary Figure S1). G4s regulate gene expression processes including transcriptional regulation by DNA G4 and splicing regulation by RNA G4 (rG4) (24–30). The involvement of DNA G4s in DNA methylation has also been reported (31). However, the relationship between rG4 and epitranscriptome is unknown. Considering the aforementioned co-localization of m6A and G4-forming sequences shown by bioinformatics analyses, rG4 might be involved in the RNA methylation process. If so, it would not only reveal a new mechanism of m6A installation dependent on the intrinsic sequence preference of METTL3/METTL14 but also provide insights into the role of rG4 in epitranscriptomic regulation.

**Figure 1.**
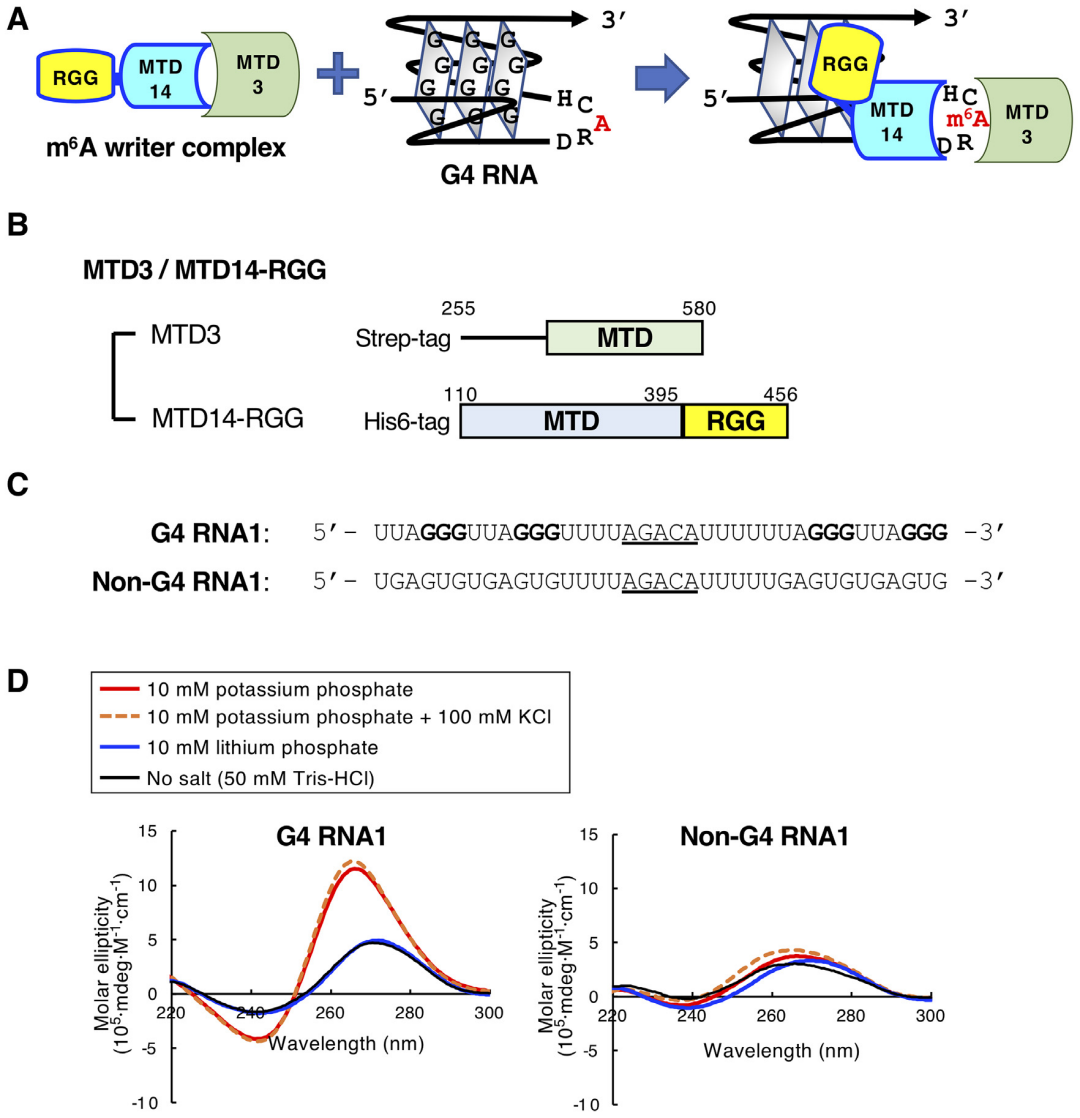
The RNA methyltransferase construct and RNAs used in this study. (**A**) The schematic representation of G4 RNA binding by the m6A writer complex. (**B**) The schematic representation of MTD3/MTD14-RGG. (**C**) The oligonucleotide sequences of G4 RNA1 and Non-G4 RNA1 are shown. G-quadruplex-forming guanines are shown in bold. Methylation consensus sequences are underlined. (**D**) The circular dichroism spectra of G4 RNA1 (left) and Non-G4 RNA1 (right) in 10 mM potassium phosphate buffer (pH 7.5), 10 mM potassium phosphate buffer (pH 7.5) with 100 mM KCl, 10 mM lithium phosphate buffer (pH 7.5) or 50 mM Tris-HCl (pH 7.5) (No-salt) buffer.

The rG4 binding proteins play important roles in determining rG4 dynamics and rG4-dependent biological processes. The domain classification of rG4 binding proteins indicates that arginine-glycine-glycine (RGG) motifs are primarily involved in the interaction with rG4s. The RGG domain is rich in arginine (R) and glycine (G) residues, typically RGG or RG arrays, and is frequently found in intrinsically disordered regions of RNA-binding proteins (32). In addition to the electrostatic interactions between RGG domains and RNA, RGG repeats of some RNA-binding proteins such as fragile X mental retardation protein and FUS/TLS are involved in the recognition of rG4 structure (33–38). METTL14 also contains RGG repeats at the C-terminus. The RNA binding ability of METTL14 was considerably decreased in the deletion mutant of the METTL14 RGG domain, thus indicating that METTL14 RGG repeats are important for RNA binding (14,16). However, the RNA binding specificity of METTL14, especially toward rG4 sequences, is elusive. In this study, we revealed that the methyltransferase METTL3/METTL14 heterodimer preferentially binds to rG4 structures and methylated adenosines in the DRACH consensus sequences close to the rG4 structures (Figure 1A). Our results suggest a possible process by which METTL3/METTL14 is directly recruited to specific methylation sites, especially near the G4-forming regions. This study provides insights into the role of rG4, a type of non-canonical structured RNAs, in epitranscriptomic regulation.

## MATERIALS AND METHODS

### Chemicals and oligonucleotides

Reagents were obtained from Sigma-Aldrich and FUJIFILM Wako Chemicals unless otherwise specified. The enzymes used for vector constructions were obtained from NEB. MazF (MazF mRNA interferase) was obtained from Takara-Bio. DNA primers for plasmid constructions were obtained from Eurofin or Thermo Fischer. Custom RNA oligonucleotides were purchased from Japan Bio Service. HeLa total RNA was extracted from HeLa cells using NucleoSpin RNA Plus (Takara-Bio).

### Protein preparation

The *Escherichia coli* BL21(DE3) cells (Nippon Gene) transformed with the MTD3/MTD14-RGG expression vector (39) (= Strep tag II-METTL3(255–580)/hexa-histidine tag-METTL14(110–456) in the pETDuet vector) (39) or MTD3/MTD14 (= Strep tag II-METTL3(255–580)/hexa-histidine tag-METTL14(110–413) in the pETDuet vector) were incubated in LB medium at 37°C, treated with 0.1 mM IPTG and 0.1 mM ZnCl_2_ at OD_600_ = ∼0.6, and further incubated at 18°C. The soluble fraction was purified using the HisTrapFF column followed by StrepTrapHP column (GE Healthcare). The following buffers were used: for the HisTrapFF column, binding buffer: 10 mM potassium phosphate, 300 mM KCl, 20 mM imidazole (pH 7.5), wash buffer: 10 mM potassium phosphate, 500 mM KCl and 20 mM imidazole (pH 7.5), elution buffer: 10 mM potassium phosphate, 300 mM KCl and 250 mM imidazole (pH 7.5). For the StrepTrapHP column, binding and wash buffer: 10 mM potassium phosphate and 100 mM KCl (pH 7.5), elution buffer: 10 mM potassium phosphate, 100 mM KCl and 2.5 mM desthiobiotin (pH 7.5). The eluted proteins were concentrated, and the buffer was replaced with 10 mM potassium phosphate (pH 7.5) by ultrafiltration. The proteins used for experiments under lithium ions were purified in the same way but using lithium phosphate and LiCl instead of potassium phosphate and KCl, respectively. The purified proteins were kept on ice and used for experiments on the same day.

The *E. coli* BL21(DE3) cells transformed with the GST-RGG expression vector (GST-RGG(396–456) in the pGEX6p-2 vector (GE Healthcare)) were incubated in LB medium at 37°C, treated with 0.1 mM IPTG at OD_600_ = ∼0.6, and further incubated at 18°C. The soluble fraction was purified using the GSTrap FF column (Cytiva) using binding buffer [50 mM Tris-HCl (pH7.5), 500 mM NaCl, and 1 mM dithiothreitol (DTT)] and elution buffer [50 mM Tris-HCl (pH8.0), 150 mM NaCl, 10 mM reduced glutathione and 1 mM DTT]. The eluted proteins were concentrated, and the buffer was replaced with 25 mM Tirs-HCl (pH 7.5) and 1 mM DTT by ultrafiltration. The purified GST-RGG protein was kept on ice and used for experiments on the same day.

### His_6_-tag pull-down and western blotting

To confirm the formation of the heterodimer between MTD3 with Strep-tag II and MTD14-RGG with His_6_-tag, 500 ng of the purified MTD3/MTD14-RGG protein was incubated with 10 μl of Ni-nitrilotriacetic acid (NTA) agarose beads (QIAGEN) in 10 mM potassium phosphate for 30 min at 4°C. The beads were precipitated by centrifugation, washed with binding buffer and eluted by elution buffer including 250 mM imidazole. The input sample and the pulled-down sample were separated by 8% SDS-PAGE. The loaded proteins were transferred onto a membrane using Trans-Blot Turbo Transfer System (Bio-Rad). The membrane was probed using a 1:5000 dilution of anti-His-tag mAb OGHis (MBL), followed by a 1:10 000 dilution of goat anti-mouse IgG antibodies (HRP) (GeneTex). Bands were visualized using ECL Prime Western Blotting Detection System (Cytiva). After strippingt the antibodies by Restore PLUS Western Blot Stripping Buffer (Thermo Fisher Scientific), the membrane was probed using a 1:5000 dilution of anti-Strep-tag II mAb 4F1 (MBL), followed by a 1:10 000 dilution of HRP-conjugated anti-mouse IgG, and then visualized.

### RNA preparation

Synthesized RNA oligonucleotides were dissolved in buffer, heated at 95°C for 5 min followed by gradual cooling to room temperature, and kept on ice until further use. For electrophoretic mobility shift assay (EMSA) and methylation assay in the presence of K^+^, 10 mM potassium phosphate buffer (pH 7.5) was used. For EMSA in the presence of Li^+^, 10 mM lithium phosphate buffer (pH 7.5) was used. For circular dichroism (CD) measurements, 100 mM KCl-containing 10 mM potassium phosphate (pH 7.5) and 50 mM Tris-HCl (pH 7.5) ( = No-Salt buffer) were also used in addition to the aforementioned buffers.

### CD measurements

Folding of annealed RNA oligonucleotides (4 μM) was confirmed using J-820-L CD spectrometer (JASCO). CD was measured using a 0.1 cm path length cell, with a scanning speed of 100 nm/min, and a response time of 0.5 s over a wavelength range of 200–300 nm. For CD measurement of the complex of RNA and MTD3/MTD14-RGG, 2 μM annealed RNA oligonucleotides and 1 or 2 μM MTD3/MTD14-RGG were mixed in 10 mM potassium phosphate buffer.

### EMSA

Serially diluted MTD3/MTD14-RGG was incubated with 2.5 nM annealed 5′FAM-labeled RNA oligonucleotides in binding buffer (10 mM potassium phosphate [pH 7.5], 25 μM ZnCl_2_, 10 μM S-adenosylmethionine (SAM), 0.5 mM DTT, 0.005% Tween 20, and 2.5% ficoll) in the presence of 50 ng/μL of yeast tRNA (Thermo Fisher Scientific) at 4°C for 1 h. For the assays in the presence of Li+, 10 mM lithium phosphate (pH 7.5) was used instead of potassium phosphate. For EMSA of the GST-RGG protein, 25 mM Tris-HCl (pH7.5) containing 10 mM KCl or 10 mM LiCl was used instead of potassium phosphate or lithium phosphate. A part of the reaction mixture was loaded onto a 5% native-polyacrylamide gel (polyacrylamide/bis-acrylamide = 19/1) and electrophoresed in 0.5 X TBE buffer at 4°C. After electrophoresis, FAM-labeled RNAs were detected using the Typhoon scanner RGB system (GE Healthcare). The peak intensity for bound and free labeled-RNA was measured using the ImageQuant software (GE Healthcare) and the fractions of bound RNA were plotted against the protein concentrations. The apparent dissociation constants (*K*_D_) were calculated by fitting the plots to the following equation using the Kaleida Graph:

$$\begin{equation*}\\\theta= {\rm{ }}\left( {\left( {\left[ P \right] + \left[ R \right] + {K_D}} \right) - {{\left( {{{\left( {\left[ P \right] + \left[ R \right] + {K_D}} \right)}^2} - 4\left[ P \right]\left[ R \right]} \right)}^{0.5}}} \right)/2\left[ R \right]\end{equation*}$$

Where [*P*] is the total protein concentration, [*R*] is the total RNA concentration and *θ* is the fraction of RNA bound to protein. The mean and standard deviation were obtained from at least three independent experiments.

### RNA methylation assay

100 or 900 nM MTD3/MTD14-RGG was mixed with 100 nM G4 RNA1 or Non-G4 RNA1, respectively, in the methylation buffer (10 mM potassium phosphate [pH 7.5], 25 μM ZnCl_2_, 10 μM SAM, 0.5 mM DTT, 0.005% Tween 20, and 2.5% ficoll) in the presence or absence of 25 ng/μl HeLa total RNA. The reaction mixture was incubated at 25°C for 40 min and heated at 95°C for 3 min to inactivate the methyltransferase activity. After methylation reaction, 1 μl of the samples was subjected to MazF reaction (total 10 μl) in MazF cleavage buffer (40 mM sodium phosphate [pH 7.5], 0.01% Tween 20, 5 mM EDTA) at 37°C for 30 min, loaded onto a 7 M urea-containing 20% polyacrylamide gel, and the fraction of uncleaved RNA (methylated RNA) was measured.

For competitive methylation assays including both G4-forming RNA and Non-G4 RNA3, 100 nM MTD3/MTD14-RGG was mixed with 100 nM FAM-labeled G4-forming RNA and 100 nM TAMRA-labeled Non-G4 RNA3 in the methylation buffer with 25 ng/μl HeLa total RNA. The reaction samples were treated in the same way as above. For the detection of FAM-labeled RNA and TAMRA-labeled RNA, the gel was scanned twice using different combinations of excitation and emission filters according to the setting of the Typhoon scanner RGB system. The methylation percentage was calculated based on the band intensity as described before (39,40).

## RESULTS AND DISCUSSION

### Design of the methyltransferase construct and G4 RNA

The heterodimer consisting of a METTL3 MTD and the METTL14 MTD with RGG repeats (MTD3/MTD14-RGG) was prepared (Figure 1B and Supplementary Figure S2). Because MTD3 and MTD14 form a stable heterodimer (6), strep-tagII-fused MTD3 and His_6_-tagged MTD14-RGG proteins were co-expressed and co-purified by two-step affinity chromatography (Supplementary Figure S2A). The heterodimer formation was confirmed by a His_6_-tag pull-down followed by blotting with an anti-strep-tag II antibody (Supplementary Figure S2B).

The target RNA sequences, G4 RNA1 and Non-G4 RNA1, were designed (Figure 1C). G4 RNA1 was designed on the basis of the sequence of telomeric repeat-containing RNA (TERRA; 5′-UUAGGGUUAGGGUUAGGGUUAGGG-3′), which forms an rG4 structure in the presence of potassium ions (41). The methylation target sequence 5′-AGACA-3′ that corresponds to the consensus methylation sequence 5′-DRACH-3′ and the spacer sequences (UUUU) were inserted in the second loop region of TERRA. The Non-G4 RNA1 sequence was designed without including the G4-forming sequences and the base composition of G4 RNA1 was maintained. The circular dichroism (CD) spectra of G4 RNA1 showed positive bands near 265 nm and negative bands near 240 nm in 10 mM potassium phosphate buffer (pH 7.5) (Figure 1D, left). These spectral changes indicate parallel rG4 structures (42). The addition of 100 mM KCl to 10 mM potassium phosphate buffer did not change the CD spectrum of G4 RNA1. This result showed that G4 RNA1 formed an rG4 structure even in 10 mM potassium phosphate buffer. On the other hand, no changes in the CD spectrum of G4 RNA1 were observed in 10 mM lithium phosphate buffer (pH 7.5) compared with that in no-salt buffer. These results showed that G4 RNA1 forms an rG4 structure in the presence of potassium ions but not lithium ions, which is in agreement with a previous report (41). No changes were observed in the CD spectra of Non-G4 RNA1 in the presence of either potassium ions or lithium ions (Figure 1D, right). This indicated that Non-G4 RNA1 does not form an rG4 structure. Unless otherwise noted, RNA oligonucleotides annealed in 10 mM potassium or lithium phosphate buffer (pH 7.5) were used in the subsequent experiments.

### Specific binding of MTD3/MTD14-RGG to G4-forming RNAs

**Figure 2.**
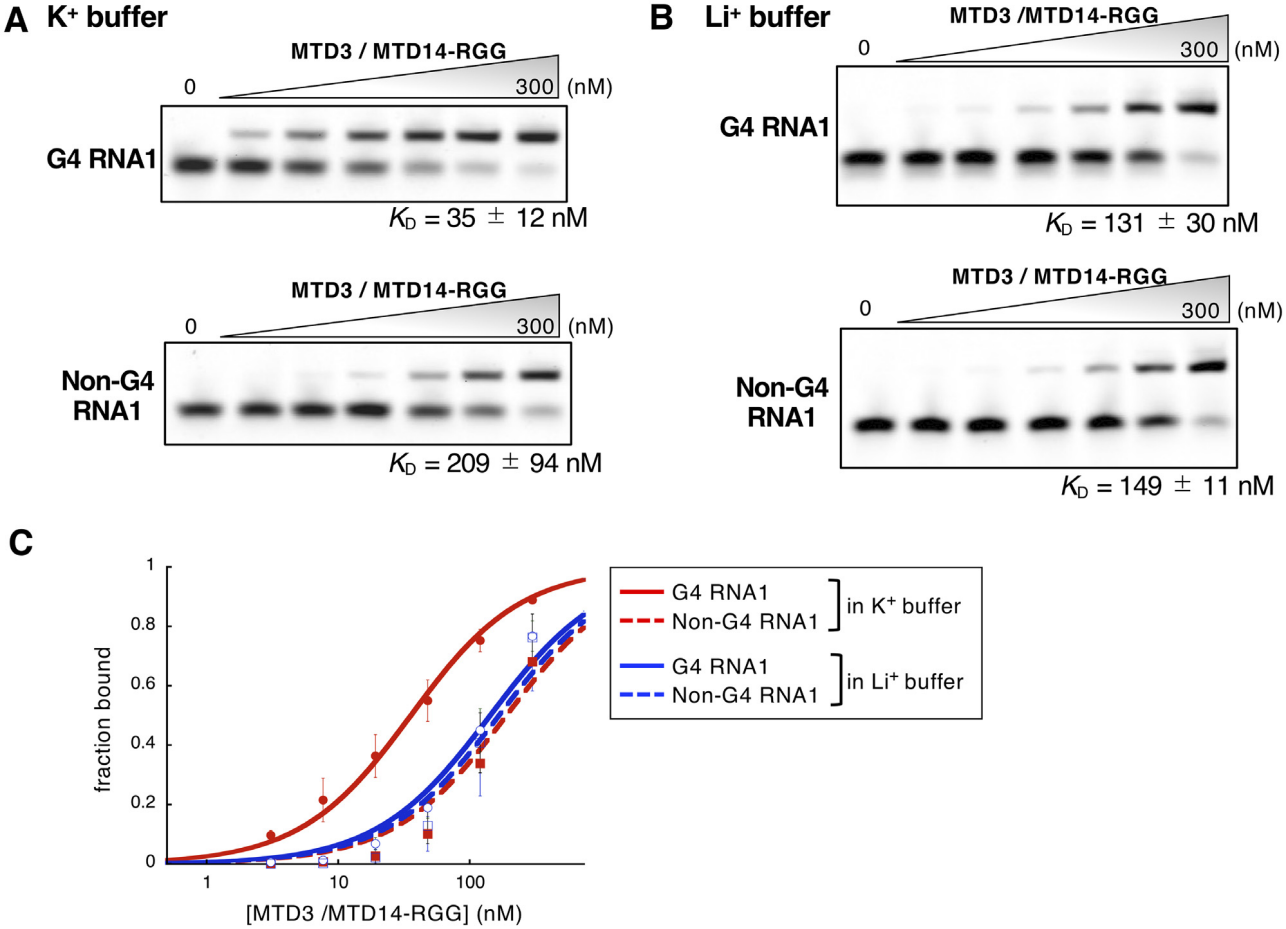
Specific interactions between the G-quadruplex structure of G4 RNA1 and MTD3/MTD14-RGG. (**A** and **B**) Representative results of the electrophoretic mobility shift assay (EMSA) of MTD3/MTD14-RGG and G4 RNA1 (upper), and Non-G4 RNA1 (lower) in 10 mM potassium phosphate (K^+^) buffer (A) or 10 mM lithium phosphate (Li^+^) buffer (B) in the presence of yeast tRNA. The concentrations of MTD3/MTD14-RGG are 0, 3, 8, 19, 48, 120 and 300 nM. (**C**) Analysis of EMSA data. Fractions of protein-bound RNA are plotted against MTD3/MTD14-RGG concentration.

The electrophoretic mobility shift assay (EMSA) showed that MTD3/MTD14-RGG binds to G4-forming RNA sequences with a higher affinity than that to non-G4-forming RNA sequences. The dissociation constant (*K*_D_) of MTD3/MTD14-RGG for G4 RNA1 was 35 ± 12 nM, whereas that for Non-G4 RNA1 was 209 ± 94 nM (Figure 2A,C and Table 1). MTD3/MTD14-RGG also bound to other G4-forming RNAs (G4 RNA2–4) having different loop lengths between G-tracts with *K*_D_ of 20–40 nM in K^+^ buffer, where these G4 RNA oligonucleotides formed rG4 structures (Table 1 and Supplementary Figure S3). On the other hand, the binding affinity of MTD3/MTD14-RGG to another non-G4-forming RNA, Non-G4 RNA2, having the same number and composition of nucleotides as G4 RNA3 and G4 RNA4, was low (*K*_D_ = 309 ± 85 nM) (Table 1 and Supplementary Figure S3B). Thus, MTD3/MTD14-RGG bound to G4-forming RNA sequences with 6–10-fold higher affinity than that when bound to non-G4-forming RNA sequences. Deletion of the RGG domain of METTL14 resulted in a more than 10-fold decrease in RNA binding affinity for G4-RNA1 (*K*_D_ = 419 ± 109 nM) compared to MTD3/MTD14-RGG (Supplementary Figure S3C). The result is in good agreement with previous reports showing that the RGG domain of METTL14 contributes remarkably to the RNA binding of METTL3/METTL14 (14,16). These results showed that MTD3/MTD14-RGG specifically recognizes G4-forming RNAs and the RGG domain is responsible for the strong RNA binding of METTL3/METTL14.

**Table 1.** RNA sequences of G4-forming and non-forming oligonucleotide RNAs and apparent dissociation constants of MTD3/MTD14-RGG and these RNAs in K^+^ buffer

RNA name	G4 loop pattern	nt	RNA sequence (5′ to 3′) bold: possible quadruplex forming guanines underlined: methylation substrate sequence	*K*_D_ (nM)
G4 RNA1	(3-16-3)	37	UUA**GGG**UUA**GGG**UUUUAGACAUUUUUUA**GGG**UUA**GGG**	35 ± 12
G4 RNA2	(3-3-16)	37	UUA**GGG**UUA**GGG**UUA**GGG**UUUUAGACAUUUUUUA**GGG**	36 ± 11
G4 RNA3	(3-8-3)	41	UUUUUUUUUUUUUUA**GGG**UUA**GGG**AGACAUUA**GGG**UUA**GGG**	29 ± 13
G4 RNA4	(3-3-3)	41	UUA**GGG**UUA**GGG**UUA**GGG**UUA**GGG**AGACAUUUUUUUUUUUU	35 ± 9
Non-G4 RNA1	(-)	37	UGAGUGUGAGUGUUUUAGACAUUUUUGAGUGUGAGUG	209 ± 94
Non-G4 RNA2	(-)	41	UUUUUUUUUUUUUGAGUGUGAGUGAGACAUGAGUGUGAGUG	309 ± 85

### Recognition of the RNA G4 structure by MTD3/MTD14-RGG

The specificity of MTD3/MTD14-RGG for the rG4 structure was demonstrated by comparing the results of EMSA in K^+^ and Li^+^ buffers. In Li^+^ buffer, G4 RNA1 did not form an rG4 structure (Figure 1D) and MTD3/MTD14-RGG bound to G4 RNA1 and Non-G4 RNA1 with comparable binding affinities (*K*_D_ = 131 ± 30 nM and 149 ± 11 nM, respectively) (Figure 2B,C). This result showed that MTD3/MTD14-RGG cannot distinguish between G4 RNA1 and Non-G4 RNA1 in Li^+^ buffer. In contrast, MTD3/MTD14-RGG bound more strongly to G4 RNA1 than Non-G4 RNA1 in K^+^ buffer. This indicated that MTD3/MTD14-RGG specifically recognizes the rG4 structure, rather than simply recognizing the interspaced repeats of guanine clusters.

The RNA binding preference of the RGG domain of METTL14 to an rG4 structure was also shown by EMSA using the GST-fused METTL14 RGG domain (GST-RGG) (Supplementary Figure S4). GST-RGG bound to G4 RNA1 stronger than to Non-G4 RNA1 in K^+^ buffer (*K*_D_ = 67 ± 9 nM and 196 ± 26 nM, respectively). The RNA binding affinity of GST-RGG for G4 RNA1 in Li^+^ buffer (*K*_D_ = 274 ± 71 nM) was decreased compared with that in K^+^ buffer. In addition, GST-RGG did not distinguish between G4 RNA1 and Non-G4 RNA1 in Li^+^ buffer. These results showed that the RGG domain itself has an RNA binding preference for the rG4 structure.

### The structure of the G4 forming RNA complexed with MTD3/MTD14-RGG

**Figure 3.**
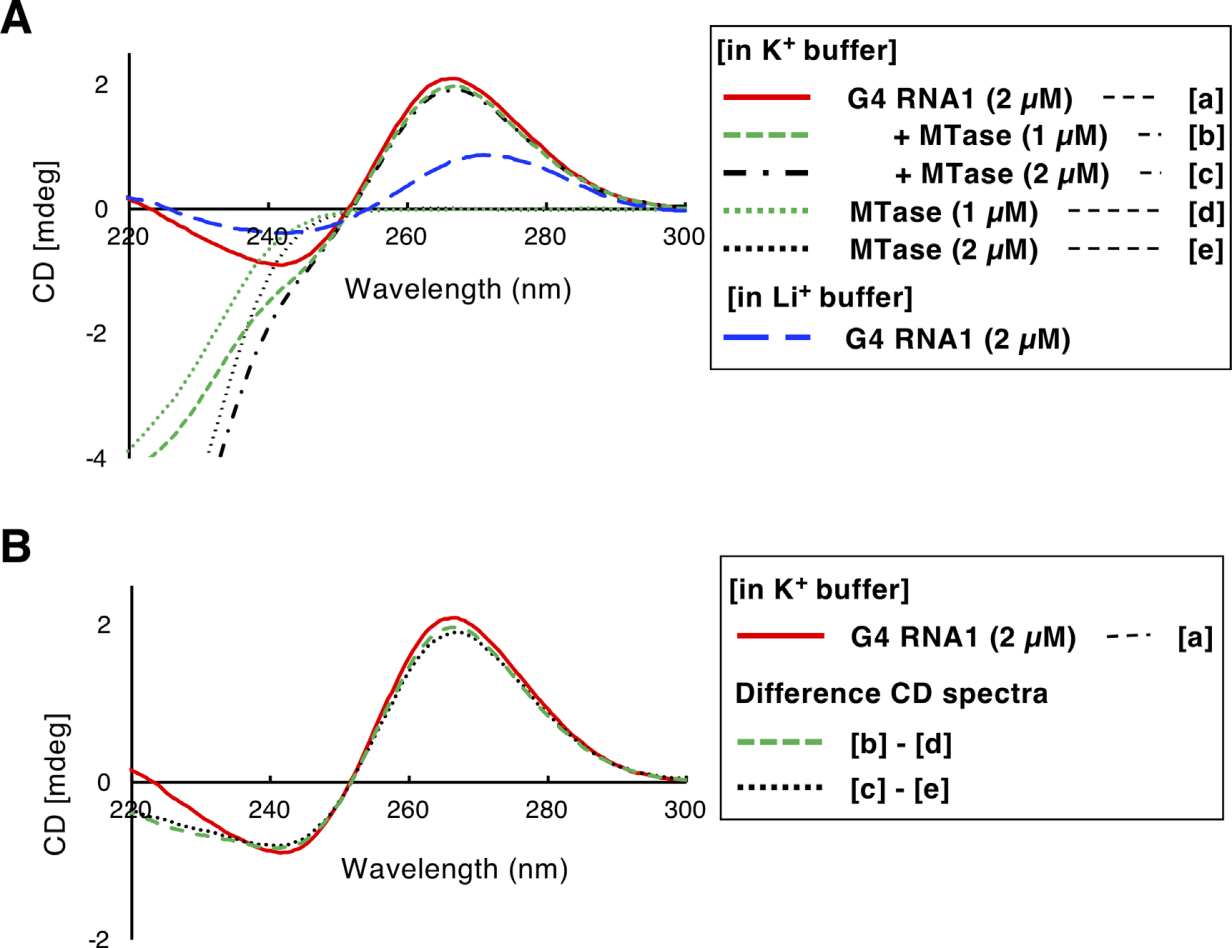
Formation of rG4 structure in free and MTD3/MTD14-RGG (MTase)-bound G4 RNA1. (A) CD spectra of 2 μM G4 RNA1 in 10 mM KP buffer ([a]; solid line) and 10 mM LiP buffer (long dashed line), 2 μM G4 RNA1 and 1 μM MTD3/MTD14-RGG mixture ([b]; dashed line), 2 μM G4 RNA1 and 2 μM MTD3/MTD14-RGG mixture ([c]; dash-dotted line), 1 μM MTD3/MTD14-RGG only ([d]; dotted line), and 2 μM MTD3/MTD14-RGG only ([e]; dotted line) in 10 mM KP buffer. (B) Difference in the CD spectra between [b] and [d] (dashed line) and between [c] and [e] (dotted line), in which CD spectra of protein only (1 or 2 μM; [d] or [e]) were subtracted from mixture of protein (1 or 2 µM) and G4 RNA1 ([b] or [c]). For a reference, the CD spectrum of 2 μM G4 RNA1 ([a]; solid line) was shown in both figures.

Furthermore, the rG4 structure was retained in the complex of G4 RNA1 and MTD3/MTD14-RGG. The CD spectrum of the complex of G4 RNA1 and MTD3/MTD14-RGG in K^+^ buffer was similar to that of G4 RNA1 alone in K^+^ buffer at wavelengths ranging from 250 to 300 nm, where protein only samples showed no peaks, regardless of the concentration of MTD3/MTD14-RGG (Figure 3A). The spectra were completely different from that of G4 RNA1 in Li + buffer. The difference CD spectra were obtained by subtracting the CD spectra of 1 or 2 μM MTD3/MTD14-RGG from the CD spectra of the mixture of G4 RNA1 and MTD3/MTD14-RGG (1 or 2 μM) (Figure 3B). The two typical peaks for the parallel rG4 structure, a positive peak around 265 nm and a negative peak around 240 nm, were almost unchanged in the presence of MTD3/MTD14-RGG. Considering the binding affinity of MTD3/MTD14-RGG for G4 RNA1 and their molar ratios, almost all RNA molecules were bound by MTD3/MTD14-RGG in the mixture of G4 RNA1 (2 μM) and MTD3/MTD14-RGG (2 μM), and less than half of RNAs were protein-bound forms in the mixture of G4 RNA1 (2 μM) and MTD3/MTD14-RGG (1 μM). Thus, the binding of MTD3/MTD14-RGG to G4 RNA1 did not change the RNA structure in K^+^ buffer. The results showed that the rG4 structure of G4 RNA1 was retained even in the complex with MTD3/MTD14-RGG.

### The methylation specificity of MTD3/MTD14-RGG for G4-RNA

The methylation specificity of MTD3/MTD14-RGG for G4-RNA1 over Non-G4 RNA1 was demonstrated by assessing the sensitivity to nonspecific RNA molecules for G4-RNA1 or Non-G4 RNA1 methylation (Supplementary Figures S5 and S6). The methylation percentages of G4 RNA1 and Non-G4 RNA1 were evaluated using the m6A-sensitive endoribonuclease, MazF, following the methylation reaction (Supplementary Figure S5 and S6) (39). The RNA methylation efficiency of METTL3/METTL14 generally depends on the substrate and the surrounding RNA sequences and structures (43). We used 100 and 900 nM MTD3/MTD14-RGG for the methylation of G4-RNA1 and Non-G4 RNA1, respectively, wherein ∼70% of the substrate RNAs were methylated in the absence of nonspecific RNAs. G4 RNA1 methylation was not significantly affected by the addition of nonspecific RNAs, i.e., total RNA extracted from HeLa cells, which was approximately 20 times the amount of G4 RNA1 (Supplementary Figure S6A,B). On the other hand, the addition of total RNA extracted from HeLa cells dramatically reduced the rate of methylated Non-G4 RNA1 (Supplementary Figure S6A,C). Thus, we found that MTD3/MTD14-RGG specifically methylates G4 RNA1, even in the presence of miscellaneous RNA.

**Figure 4.**
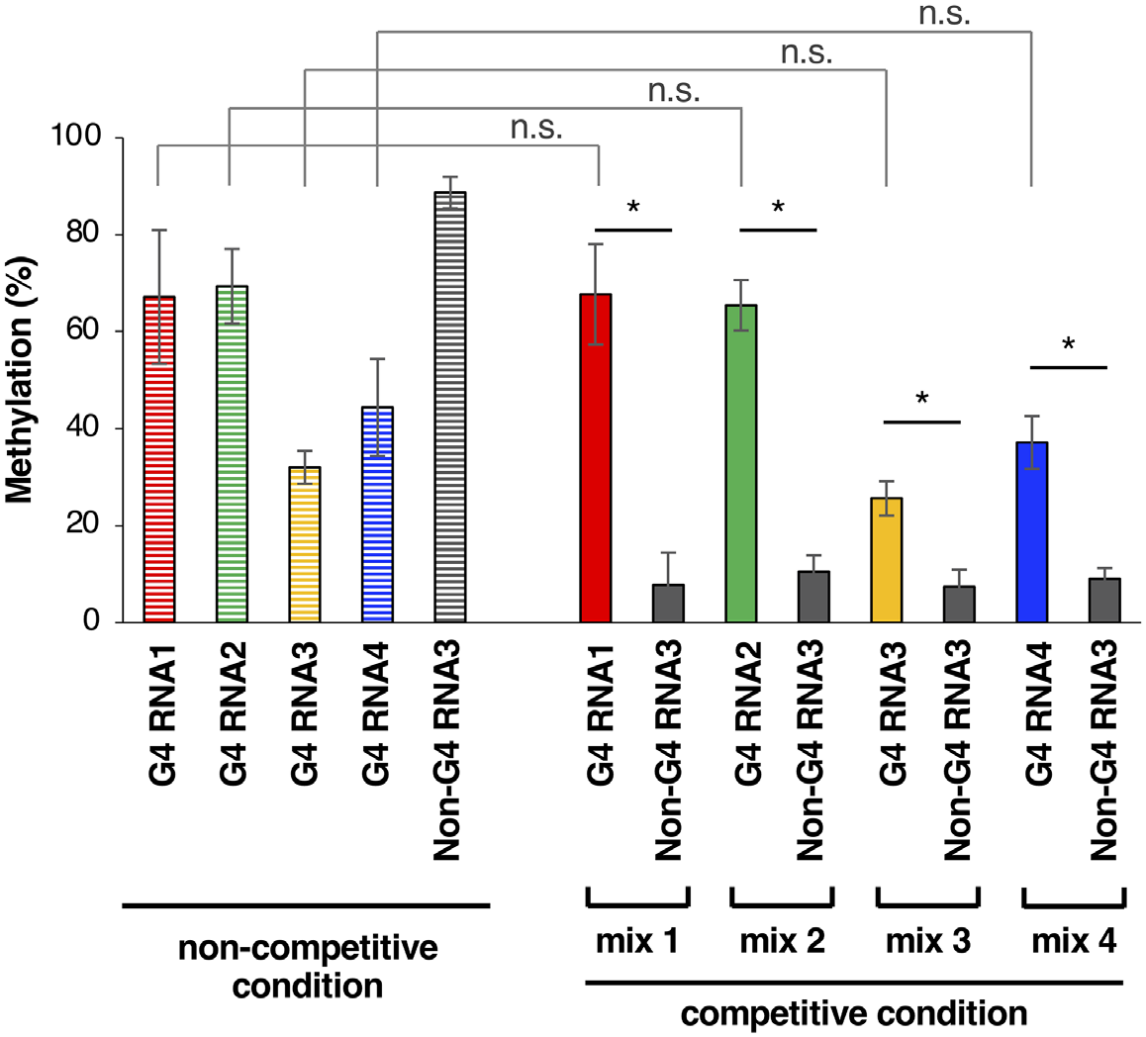
Preferential methylation of G4-forming oligonucleotide RNA by MTD3/MTD14-RGG. The methylation rates of G4 RNA1–4, and Non-G4 RNA3 (5′- UGAGUGUUAGGGACAUGAGUGUGAGUG-3′) by 100 nM MTD3/MTD14-RGG for 60 min in non-competitive conditions are shown on the left side (border color). The methylateion percentages of G4 RNA1–4 and Non-G4 RNA3 in the mixture of one of the G4 RNAs and Non-G4 RNA3 in the presence of HeLa total RNA (competitive condition) are shown on the right side; *, *P*< 0.01; n.s., *P* > 0.05.

The methylation selectivity of MTD3/MTD14-RGG to DRACH sequence within or close to the G4-forming sequences was shown by competitive methylation reactions in the presence of G4-forming RNA, non-G4-forming RNA, and total RNA extracted from HeLa cells (Figure 4). Under noncompetitive conditions, the methylation percentages of G4 RNA1–4 (Table 1 for sequence) by 100 nM MTD3/MTD14-RGG ranged from 30% to 70% depending on the RNA sequence. We used Non-G4 RNA3 (5′-UGAGUGUUAGGGACAUGAGUGUGAGUG-3′) as the non-G4-forming RNA that contained GGACA sequence instead of AGACA as a methylation substrate and was easily methylated (∼90%) by 100 nM MTD3/MTD14-RGG under non-competitive conditions in contrast to Non-G4 RNA1. By using a FAM-labeled G4-forming RNA and TAMRA-labeled non-G4-forming RNA, the methylation percentages of each of the two types of RNAs coexisting in the reaction mixture were measured simultaneously (Supplementary Figures S5C and S7) (40). As shown in Figure 4, the preferential RNA methylation of G4-forming RNAs was achieved in the mixture of G4-forming RNA, Non-G4 RNA3, and HeLa total RNA. Even though the methylation percentage of Non-G4 RNA3 was the highest under non-competitive conditions, the methylation percentage of G4-forming RNAs was significantly higher than that of Non-G4 RNA3 under competitive conditions in all mixtures 1–4, regardless of the positions of the methylation consensus sequence located inside the loop (G4 RNA1–3) or outside the G4 structure (G4 RNA4). Furthermore, the methylation percentage of G4-forming RNAs in competitive conditions did not significantly change compared with that in non-competitive conditions. Namely, competitors did not hamper the methylation of G4-forming RNAs. In contrast, the methylation percentage of Non-G4 RNA3 was greatly reduced to 10% or less when coexisting with the G4-forming RNAs and total RNA extracted from HeLa cells. These results showed that MTD3/MTD14-RGG specifically methylated adenosines close to the G4-forming sequences under competitive conditions, whereas the methylation of Non-G4 RNA3 was strongly reduced by the coexistence of miscellaneous RNA.

Sequencing analyses of immunoprecipitated RNA by anti-m6A antibodies have shown that the consensus substrate sequence for adenosine methylation is 5′-DRACH-3′ (8, 9). However, the methylation efficiency differs among the DRACHs present in consecutive sequences in *in vitro* methylation experiments performed using METTL3/METTL14 (43–45). The surrounding sequences and the structure of the substrate DRACHs can change the mode of interactions between substrate adenosine and the catalytic core including CCCH-type zinc finger domains of METTL3 and positively charged residues located at MTD3 and MTD14, resulting in RNA-dependent differences in methylation efficiency, although the details of substrate recognition are still unknown (5,44,46). In our experiments, the methylation percentage of G4 RNA3 was about half of the methylation percentage of G4 RNA1 or G4 RNA2 (Figure 4), despite the comparable dissociation constants of MTD3/MTD14-RGG for G4 RNA1∼4 (Table 1). The location of the substrate RNA core is a plausible reason for the low methylation efficiency of G4 RNA3, in which the AGACA sequence is at the second loop region with no spacer sequences (Table 1). The relationship between rG4 binding by the RGG domain of METTL14 and catalytic reaction by core methyltransferase domains needs to be further examined in the future. Nonetheless, the key points are as follows: (1) RGG domain of METTL14 can contribute to guide the methyltransferase core region to substrate DRACH sequences close to rG4 sites and (2) RNA methylation close to the G4-forming sequences was not affected even in a competitive condition.

In this study, the importance of RGG repeats of METTL14 for RNA binding of the METTL3/METTL14 heterodimer was investigated. In particular, the RNA binding preference to the rG4 structure was reported for the first time. This was shown by the higher affinity of MTD3/MTD14-RGG to G4-forming RNAs compared with Non-G4 RNAs in the presence of potassium ions, wherein G4-forming RNAs formed an rG4 structure. The fact that MTD3/MTD14-RGG had comparable binding affinities for G4 RNA1 and Non-G4 RNA1 in the presence of lithium ions that do not induce an rG4 structure to G4 RNA1 supported the RNA binding preference of MTD3/MTD14-RGG for an rG4 structure. In addition, the methylation of G4-forming RNAs selectively occurred in the mixtures of G4-forming RNAs and non-G4-forming RNAs in the presence of total RNA extracted from HeLa cells. It is plausible that the preferential binding of the RGG repeats to the rG4 structure contributes to the selective RNA methylation close to rG4.

Previous reports have indicated the contribution of protein–protein interactions of the m6A writer complex in determining the methylation target sites (11–16). The zinc finger domain of METTL3 is also important for RNA binding and methylation in DRACH sequences (46). A recent study by Wang *et al.* reported the importance of arginine methylation of the RGG repeat of METTL14 for the RNA binding and RNA methylation activity of METTL3/METTL14 (16). Although our precise *in vitro* study showed direct recognition of the rG4 structures by the m6A writer complex, further examinations are needed to elucidate the mechanism underlying m6A installation. The next goal is to elucidate the roles of METTL14 RGG repeats in a G4-dependent RNA methylation within cells. Considering the bioinformatics analyses of co-localization of G4-forming sequences in viral RNA (ZIKV and HIV) and at human pre-mRNA intron splice sites (20,21), we believe that our findings suggest the existence of a new process for recruiting METTL3/METTL14 to specific methylation sites close to G4-forming regions among many DRACH sequences.

In the case of DNA CpG methylation, Mao *et al.* reported that DNA methyltransferase 1 exhibits a higher binding affinity for G4-structured DNA than that of duplex, hemimethylated, or single-stranded DNA, suggesting the involvement of G4 DNA in epigenetic regulation (31). Pandolfini *et al.* reported that methyltransferase METTL1 catalyzes the *N*7-methylation of guanosine within rG4 in pri-miRNA (47). Our study is the first to report the RNA binding preference of the m6A writer complex for the rG4 structure and provides new perspectives on the biological role of G4-quadruplex RNA in epitranscriptomic regulation.

## DATA AVAILABILITY

All data and materials are available upon request.

## Supplementary Material

gkab1211_Supplemental_File
