# Supplementary material for: Recognition of G-quadruplex RNA by a crucial RNA methyltransferase component, METTL14
Source: Nucleic Acids Res. 2021 Dec 15;50(1):449–57. doi: 10.1093/nar/gkab1211 (PMC8755082; doi:10.1093/nar/gkab1211)
Supplement: gkab1211_Supplemental_File [file gkab1211_supplemental_file.pdf]

Supplementary data

Recognition of G-quadruplex RNA by a crucial RNA methyltransferase component, METTL14

Atsuhiro Yoshida [1], Takanori Oyoshi [2], Akiyo Suda [1], Shiroh Futaki [1] and Miki Imanishi [1,*]

[1] Institute for Chemical Research, Kyoto University, Uji, Kyoto 611-0011, Japan

[2] Graduate School of Integrated Science and Technology, Shizuoka University, Suruga-ku, Shizuoka 422-8017, Japan

Supplementary Figures (Fig S1~S7)

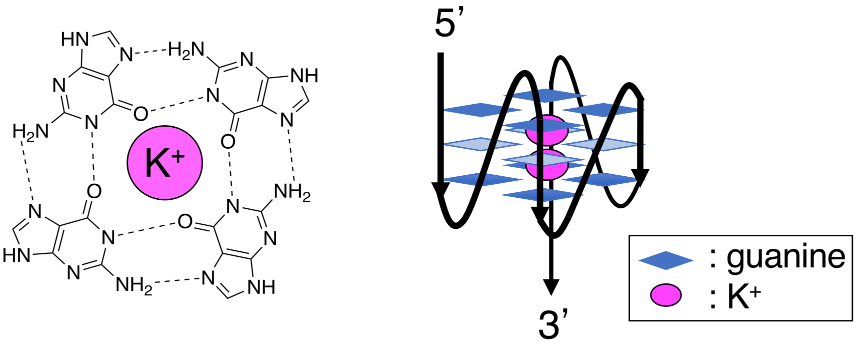

(G-tetrad)　　(RNA G-quadruplex)

Figure S1: The schematic representation of G-tetrad and RNA G-quadruplex. The G-rich sequences, 5´-$G_nL_xG_nL_xG_nL_xG_n$-3´ ($n \geq 2$, $1 \leq x \leq 20$), in which four or more runs of guanine (G) are separated by loops (L = A, G, C or U), form the RNA G-quadruplex structure stabilized by pottasium ions.

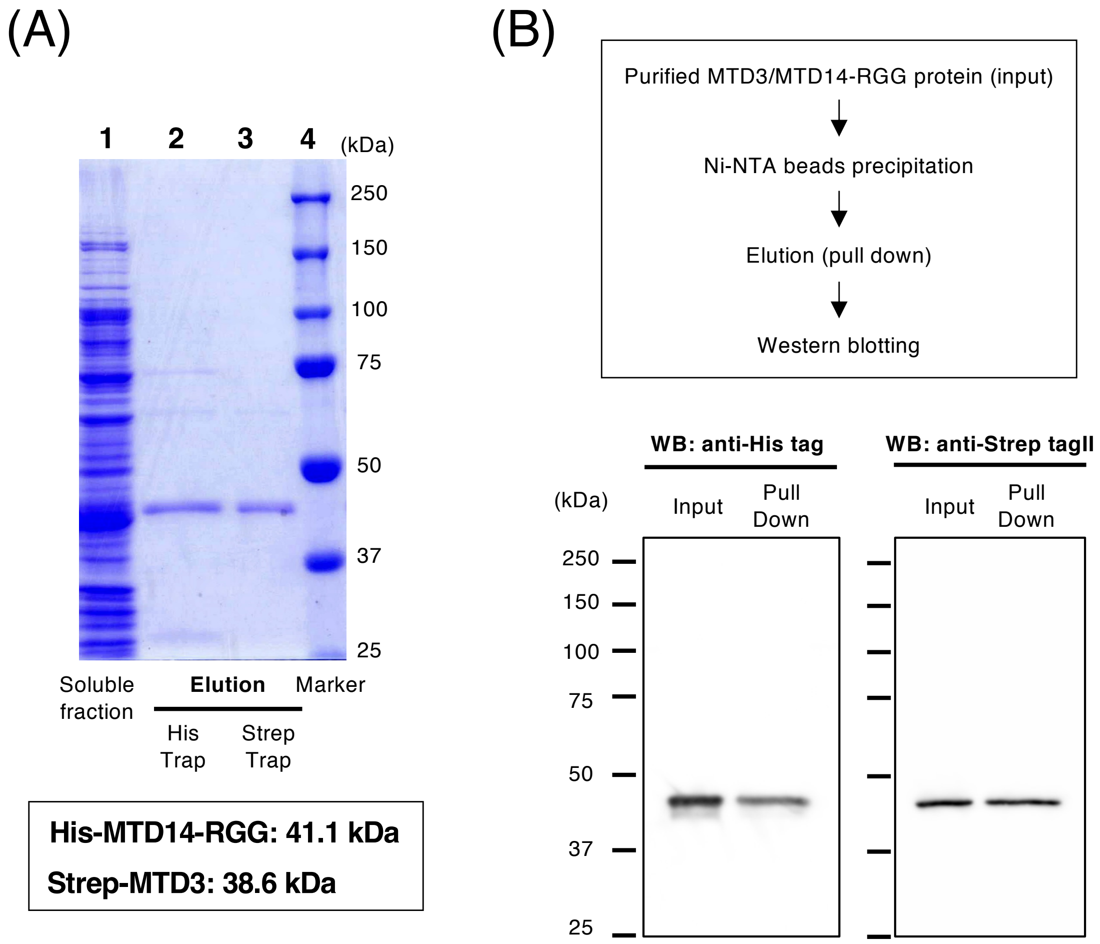

Figure S2: Confirmation of heterodimer formation of MTD3/MTD14-RGG. (A) Coomassie Brilliant Blue-stained SDS-polyacrylamide gel. Lane1-4: 1; soluble fraction of *E.coli* lysate expressing Strep-tagged MTD3 and His_6-tagged MTD14-RGG, 2; eluted sample from HisTrap column, 3; eluted sample from StrepTrap column following elution from HisTrap column, 4; molecular weight marker (Precision Plus; Bio-rad). (B) Ni-NTA-beads-pull down experiment of purified MTD3/MTD14-RGG. The input and pulled down proteins were detected by western blotting using anti-His tag antibody (left) and anti-Strep tagII antibody (right).

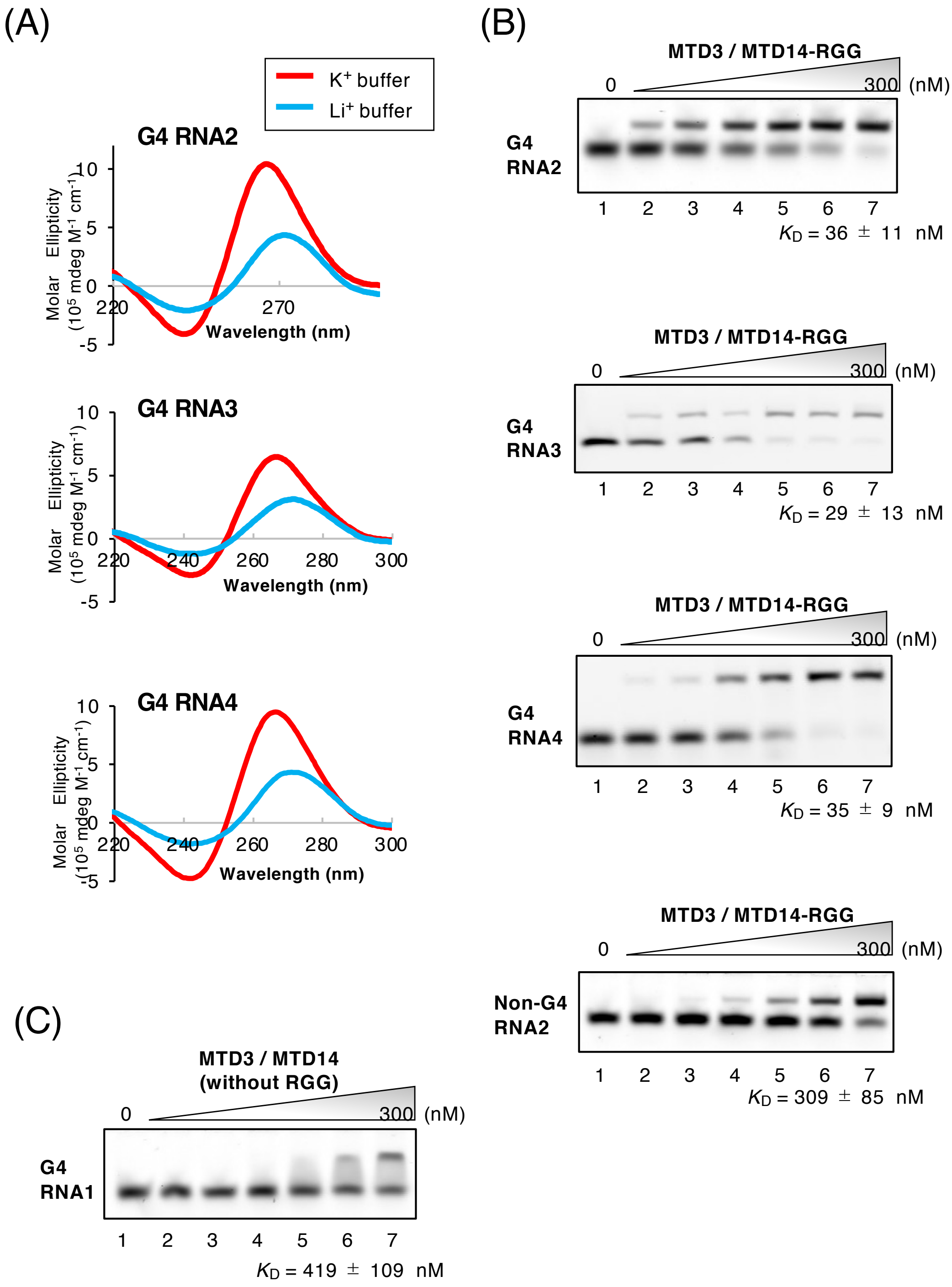

Figure S3: Preferential RNA binding of MTD3/MTD14-RGG to G4-forming RNAs. (A) CD spectra of G4 RNA2, 3, and 4 in 10 mM KP buffer (red) and 10 mM LiP buffer (blue). The CD spectra show that G4 RNA2, 3, and 4 form a G-quadruplex structure in 10 mM KP buffer. (B, C) Representative results of EMSA of MTD3/MTD14-RGG to G4 RNA2, 3, and 4 and Non-G4 RNA2 (B) and MTD3/MTD14 without RGG to G4 RNA1 (C) in the presence of yeast tRNA in 10 mM KP buffer. Lanes 1-7; 0, 3, 7.7, 19, 48, 120, and 300 nM of MTD3/MTD14-RGG (B) or MTD3/MTD14 (C).

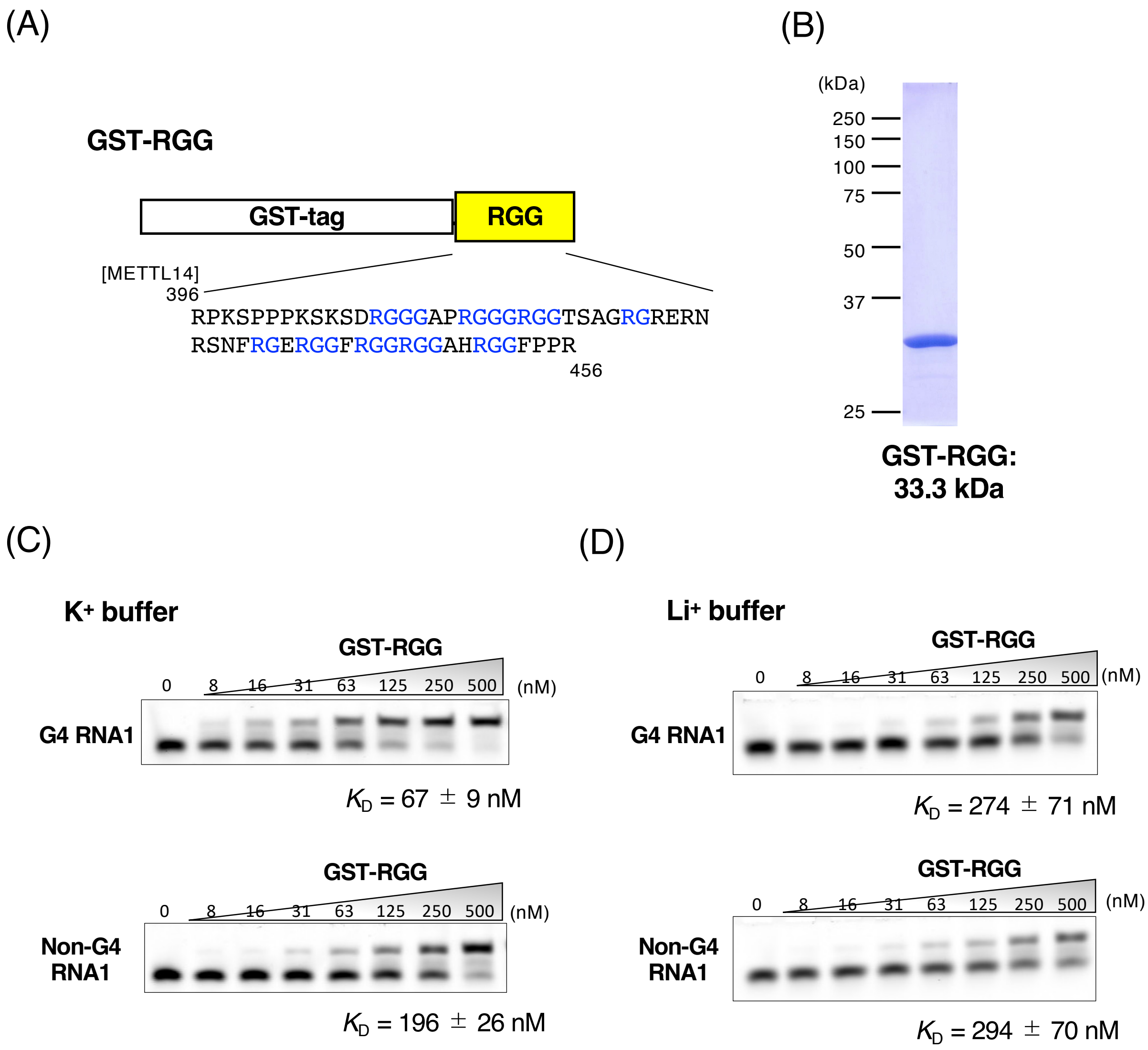

Figure. S4: Preferential RNA binding of the RGG domain to rG4 structured G4 RNA1. (A) Schematic representation of the GST-RGG construct. (B) Purified GST-RGG protein stained with CBB after SDS-PAGE. (C, D) Representative results of EMSA of GST-RGG to G4 RNA1 and Non-G4 RNA1 in the presence of 50 ng/μL yeast tRNA in 25 mM Tris-HCl (pH7.5) containing 10 mM KCl (C) or 10 mM LiCl (D). left to right; 0, 8, 16, 31, 63, 125, 250 and 500 nM of GST-RGG.

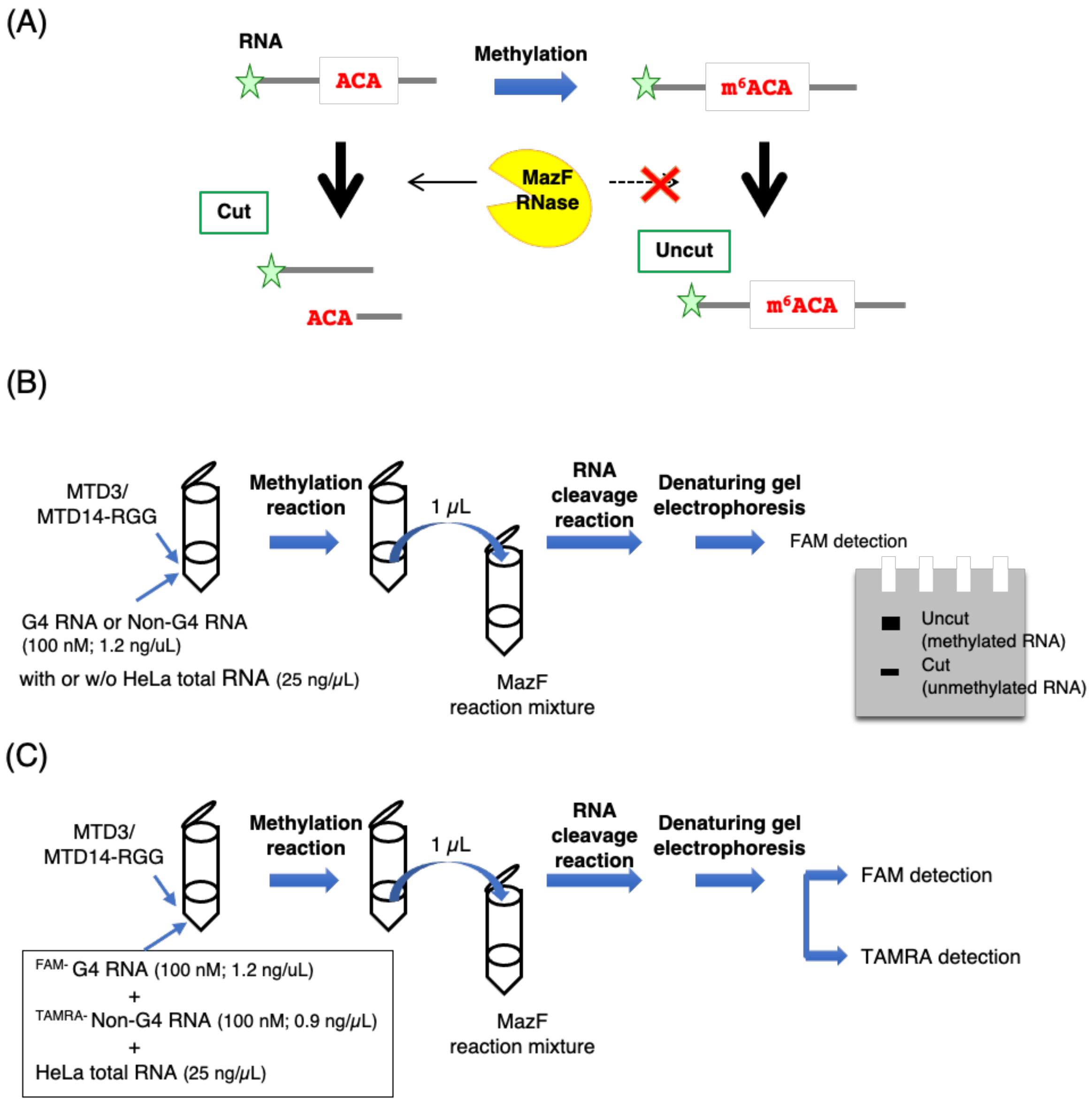

Figure S5: MazF-based methylation assay. (A) MazF is an ACA-sequence specific and (m6A)CA-sensitive endribonuclease. (B)(C) The schematic procedure of the methylation assay under noncompetitive (B) or competitive (C) condition. Substrate RNAs were incubated with MTD3/MTD14-RGG. Then, the samples were treated by MazF endoribonuclease, which cleaves only unmethylated RNA. The ratio of uncut RNA fractions indicates the methylated ratio of RNA.

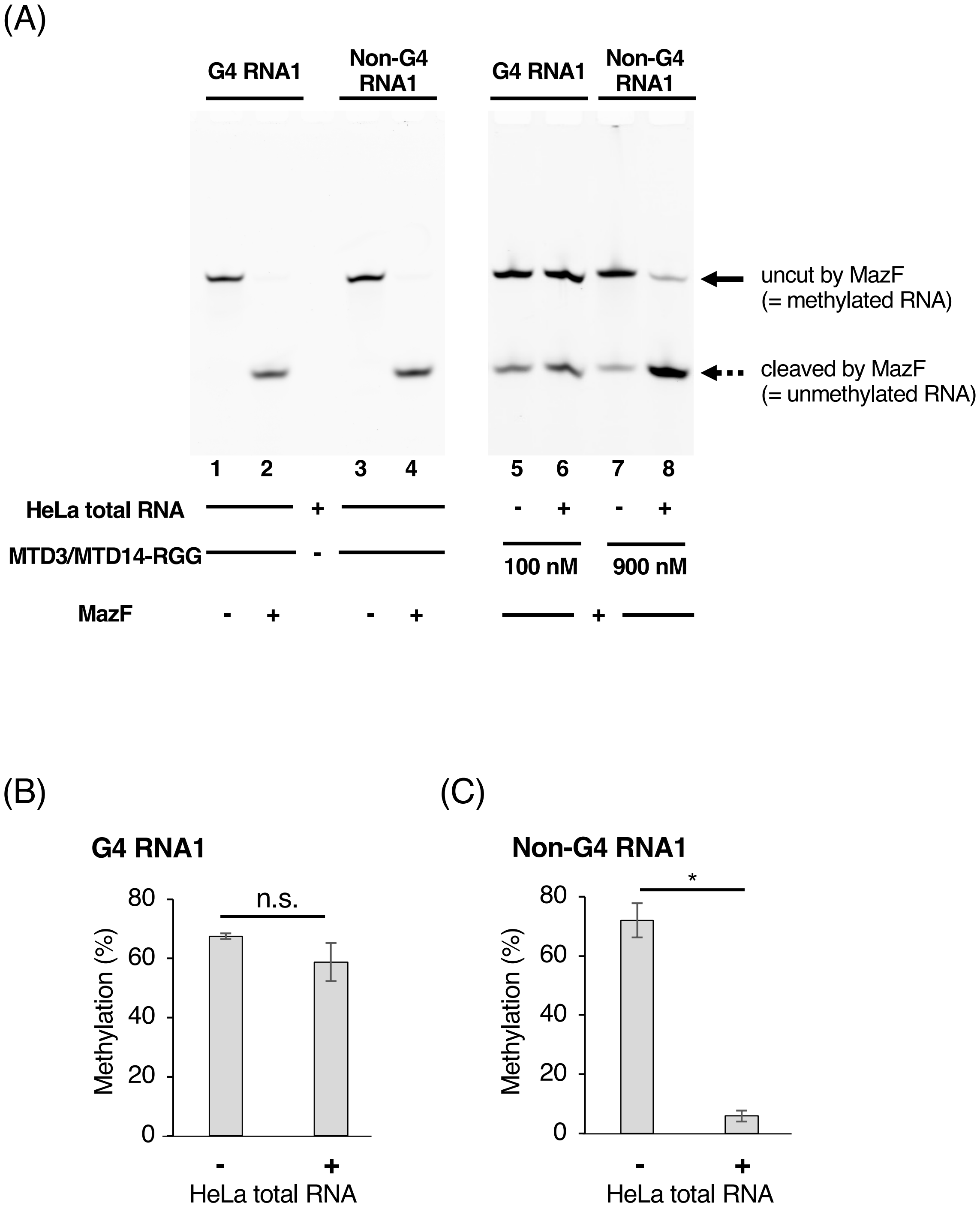

Figure S6: G4 structure-dependent RNA methylation by MTD3/MTD14-RGG in the presence of miscellaneous competitor RNA (HeLa total RNA) in 10 mM KP buffer. (A) RNA methylation analysis by denaturing polyacrylamide gel electrophoresis of MazF-treated RNA after methylation reaction of G4 RNA1 or Non-G4 RNA1 with MTD3/MTD14-RGG in the presence of absence of HeLa total RNA. The percentages of the methylated G4 RNA1 (B) or Non-G4 RNA1 (C) by 100 nM or 900 nM MTD3/MTD14-RGG, respectively are shown as the mean ± SD. The statistical analyses were performed using student-t test (*; p < 0.01, n.s.; not significant).

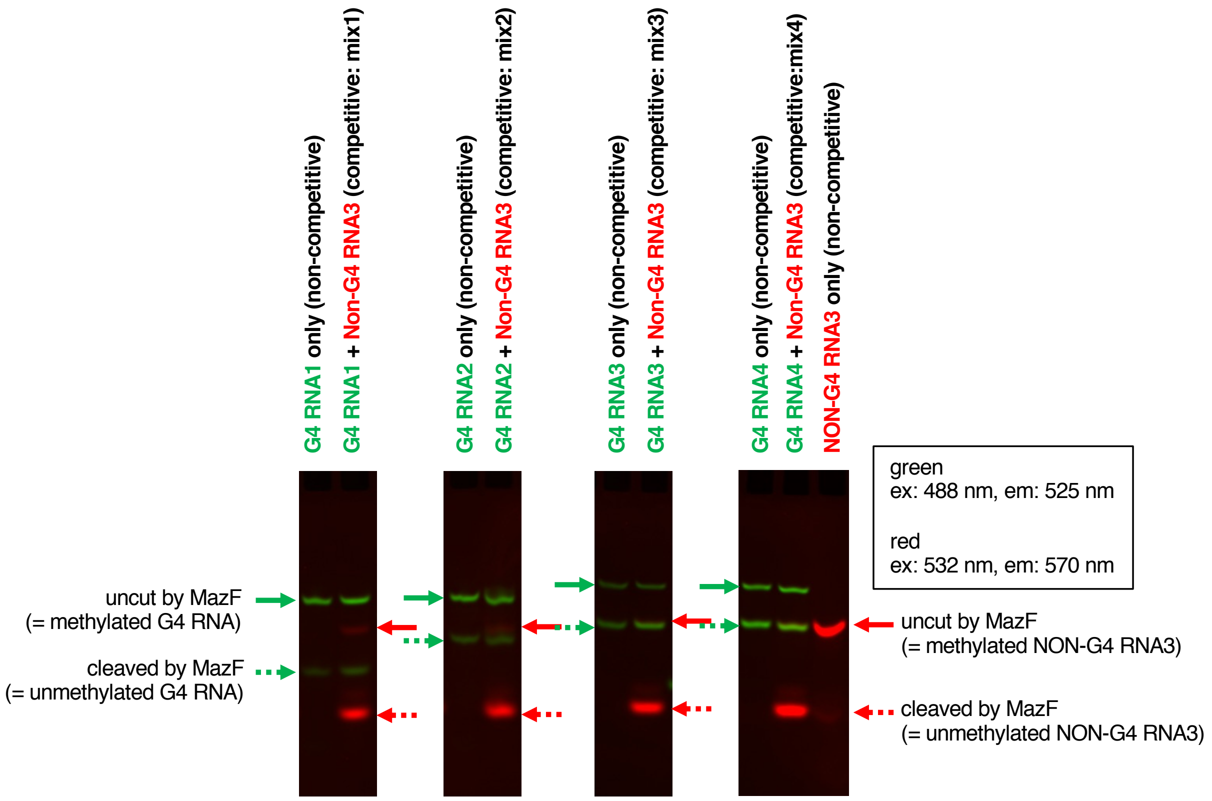

Figure S7: (related to Figure 4) Non-competitive or competitive RNA methylation analysis by denaturing polyacrylamide gel electrophoresis of MazF-treated RNA after methylation reaction by 100 nM MTD3/MTD14-RGG. One of the FAM-labeled G4 RNAs (1, 2, 3 or 4) and TAMRA-labeled Non-G4 RNA3 were mixed and reacted with MTD3/MTD14-RGG in same reaction tubes in competitive conditions. Each methylation percentage was analyzed by denaturing gel electrophoresis of RNA after MazF treatment; FAM-labeled RNA fragments and TAMRA-labeled RNA fragments were detected using the corresponding excitation and emission filters, respectively.
